# Xpert MTB/RIF on pleural biopsy in suspected pleural TB: Does it add anything other than cost?

**DOI:** 10.7196/AJTCCM.2021.v27i1.136

**Published:** 2021-03-09

**Authors:** J A Shaw, C F N Koegelenberg

**Affiliations:** 1 DST-NRF Centre of Excellence for Biomedical Tuberculosis Research; South African Medical Research Council Centre for Tuberculosis Research, Division of Molecular Biology and Human Genetics, Stellenbosch University, Cape Town, South Africa; 2 Division of Pulmonology, Department of Medicine, Stellenbosch University and Tygerberg Academic Hospital, Cape Town, South Africa

## Editorial

Pleural tuberculosis remains one of most frequent causes of pleural exudates globally and the second most common form of extrapulmonary tuberculosis.^[1]^ It presents as a spectrum disease ranging from uncomplicated effusions that resolve spontaneously to frank empyema, and is the result of an effusive Th1 lymphocyte-driven immune response to paucibacillary infection of the pleura.^[2]^

The detection of *Mycobacterium tuberculosis* in sputum, pleural fluid or tissue, either by microscopy, culture or nucleic acid amplification tests (NAATs), or histological demonstration of caseating granulomas in pleura along with acid-fast bacilli (AFB), is the current gold standard for diagnosis.^[3]^ In high-burden settings, however, the diagnosis is frequently inferred in patients who present with a lymphocytic predominant exudate and high pleural fluid adenosine deaminase (ADA) or interferon gamma (IFN-γ) levels.^[2,3]^ The latter approach has the advantage of expediting the diagnosis and has a positive predictive value (PPV) of 98%.^[4]^ However, it does not allow for drug sensitivity testing and can be false positive in other diseases, e.g. lymphoma.^[4]^ Tuberculin skin test (TST) and IFN-γ release assay (IGRA) are unfortunately not very useful in diagnosing pleural tuberculosis, as both assays have poor positive and negative predictive values.^[5,6]^ Although sputum culture is positive in up to 50% of cases, sputum Xpert MTB/RIF continues to have an uncertain yield.^[7,8]^

Microscopy for AFBs in pleural fluid is positive less than 10% of the time, and *M. tuberculosis* is cultured in less than 30% of cases when solid state media are used.^[2]^ Modern liquid culture media has a much higher yield (up to 70% in some reports), and also allows for drug susceptibility testing.^[9]^

Ultrasound-guided closed pleural biopsy has a diagnostic yield of up to 90%, and yields a positive culture in almost 80% of cases, whereas thoracoscopic pleural biopsy has a sensitivity of 100%.^[4,9,10]^ Unsurprisingly, most authorities advocate thoracoscopic pleural biopsy for pleural exudates that remain undiagnosed after thoracentesis.^[11]^

A Cochrane review published in 2018 (that included only 4 studies) concluded that Xpert MTB/RIF on pleural tissue had a pooled sensitivity of 30.5% and specificity of 97.4%.^[12]^ In the present addition, Yoo *et al*. ^[13]^ enrolled a relatively young cohort of patients with a high pre-test probability of pleural tuberculosis and performed unguided closed pleural biopsies on them. Tissue was subjected to Xpert MTB/ RIF assay in combination with routine solid medium culture, in parallel to histological analysis. The investigators reported the sensitivity and specificity of Xpert MTB/RIF to be 30% and 100%, respectively, using culture as the gold standard, and 20% and 95.7% compared with histopathology as the gold standard. ^[13]^ The authors concluded that Xpert MTB/RIF performed on pleural tissue obtained by closed pleural biopsy did not increase diagnostic yield, but it potentially shortened the time to diagnosis compared with conventional investigations.

The study by Yoo *et al*. ^[13]^ underscores the fact that Xpert MTB/RIF unfortunately has a limited role in diagnosing pleural tuberculosis. It is positive in a minority of cases, as once again shown in this study. When positive, it has the advantage of detecting rifampicin resistance, but whether it actually ‘shortens time for diagnosis’ could be debated, as pleural fluid differential cell count (more specifically the lymphocyte to neutrophil ratio) ADA and/or IFN-γ arguably has a similar turnaround time with a much higher PPV.

The ‘take-home’ message from the study by Yoo *et al*. ^[13]^ is simple: while the Xpert MTB/RIF assay has revolutionised diagnosis of pulmonary tuberculosis, its role in pleural tuberculosis remains very limited, to the point where it cannot be justified to routinely perform the test for suspected cases.
